# Dynamic X-ray elastography using a pulsed photocathode source

**DOI:** 10.1038/s41598-021-03221-y

**Published:** 2021-12-16

**Authors:** Chika Kamezawa, Avilash Cramer, Wolfgang Krull, Wataru Yashiro, Kazuyuki Hyodo, Rajiv Gupta

**Affiliations:** 1 Department of Materials Structure Science, SOKENDAI (The Graduate University for Advanced Studies), 1-1 Oho, Tsukuba, Ibaraki 305-0801 Japan; 2 Institute of Materials Structure Science, High Energy Accelerator Research Organization (KEK), 1-1 Oho, Tsukuba, Ibaraki 305-0801 Japan; 3 Institute of Multidisciplinary Research for Advanced Materials (IMRAM), Tohoku University, 2-1-1 Katahira, Aoba-ku, Sendai, Miyagi 980-8577 Japan; 4 Massachusetts Institute of Technology, Cambridge, 02139 USA; 5 Harvard Medical School, Boston, 20115 USA; 6 Massachusetts General Hospital, Boston, 02114 USA

**Keywords:** Breast cancer, Biomedical engineering, Electrical and electronic engineering

## Abstract

X-ray absorption of breast cancers and surrounding healthy tissue can be very similar, a situation that sometimes leads to missed cancers or false-positive diagnoses. To increase the accuracy of mammography and breast tomosynthesis, we describe dynamic X-ray elastography using a novel pulsed X-ray source. This new imaging modality provides both absorption and mechanical properties of the imaged material. We use a small acoustic speaker to vibrate the sample while a synchronously pulsed cold cathode X-ray source images the mechanical deformation. Using these stroboscopic images, we derive two-dimensional stiffness maps of the sample in addition to the conventional X-ray image. In a breast phantom composed of ZrO_2_ powder embedded in gel, dynamic elastography derived stiffness maps were able to discriminate a hard inclusion from surrounding material with a contrast-to-noise ratio (CNR) of 4.5. The CNR on the corresponding absorption image was 1.1. This demonstrates the feasibility of dynamic X-ray elastography with a synchronously pulsed X-ray source.

## Introduction

Tissue elastography, a noninvasive imaging modality used to assess tissue stiffness, has been under development for the past three decades^1–6^. Because cancerous lesions have different mechanical properties than adjacent healthy tissue, elastography aims to detect such lesions based on their stiffness. Even when such lesions have similar X-ray attenuation to the surrounding tissue, and are therefore not apparent on conventional mammography, elastography may be able to detect them.

There are two main classes of elastography techniques: static, and dynamic. In static elastography, a fixed static pressure is applied to the tissue under investigation and local strain from tissue deformation is mapped by imaging. This method can qualitatively evaluate the pattern of deformation and identify lesions^7–9^. Static elastography, however, does not provide a quantitative map of tissue stiffness because it lacks a direct measure of the stress field within the tissue^10^. Dynamic elastography, the topic of this paper, uses shear wave propagation to map both stress and strain in the tissue in response to dynamic mechanical deformation, producing a quantitative elasticity map.

In dynamic elastography, shear waves are generated inside a sample by a superficially applied, time varying pressure. Such a pressure could be generated using air vibration, force impulse from acoustic radiation, or other methods that impart a shear wave which travels within the tissue. The time-varying stress and strain generated by this shear wave are continuously imaged. Using the observed image sequence, a quantitative elasticity map is generated by inferring the spatial and temporal variation in the tissue displacement from the velocity of the propagating shear wave. Depending on the sample of interest, a number of different medical imaging techniques may be employed to image the shear wave. Both magnetic resonance (MR) and ultrasound (US) elastography have rapidly expanded into clinical practice and have been used for liver and breast diseases, respectively^11–13^. In recent years, elastography studies have also been reported in optical coherence tomography^14, 15^ and photoacoustic imaging^16, 17^.

Despite higher spatial resolution and superior penetration depth of X-rays compared with other imaging modalities, relatively few studies have reported on dynamic X-ray elastography. In the past decade, static elastography using X-ray imaging has been reported by Hamilton et al.^18^, Kim et al.^19, 20^, and Sutphin et al.^21^. As mentioned before, these static techniques do not provide a quantitative elasticity map. We recently reported on dynamic X-ray elastography^22^ that provides a two-dimensional map of storage and loss moduli. This prior study used a continuous X-ray source that was divided into individual pulses using an optical chopper wheel, an arrangement that was somewhat cumbersome to implement and difficult to accurately time. In this paper, we demonstrate the feasibility of dynamic X-ray elastography that obviates the need for a chopper wheel by synchronously pulsing the X-ray source.

X-ray elastography could improve the diagnostic accuracy of mammography and other X-ray imaging techniques, and reduce unnecessary biopsies and other procedures^23^.

## Materials and methods

### Experimental setup

**Figure 1:**
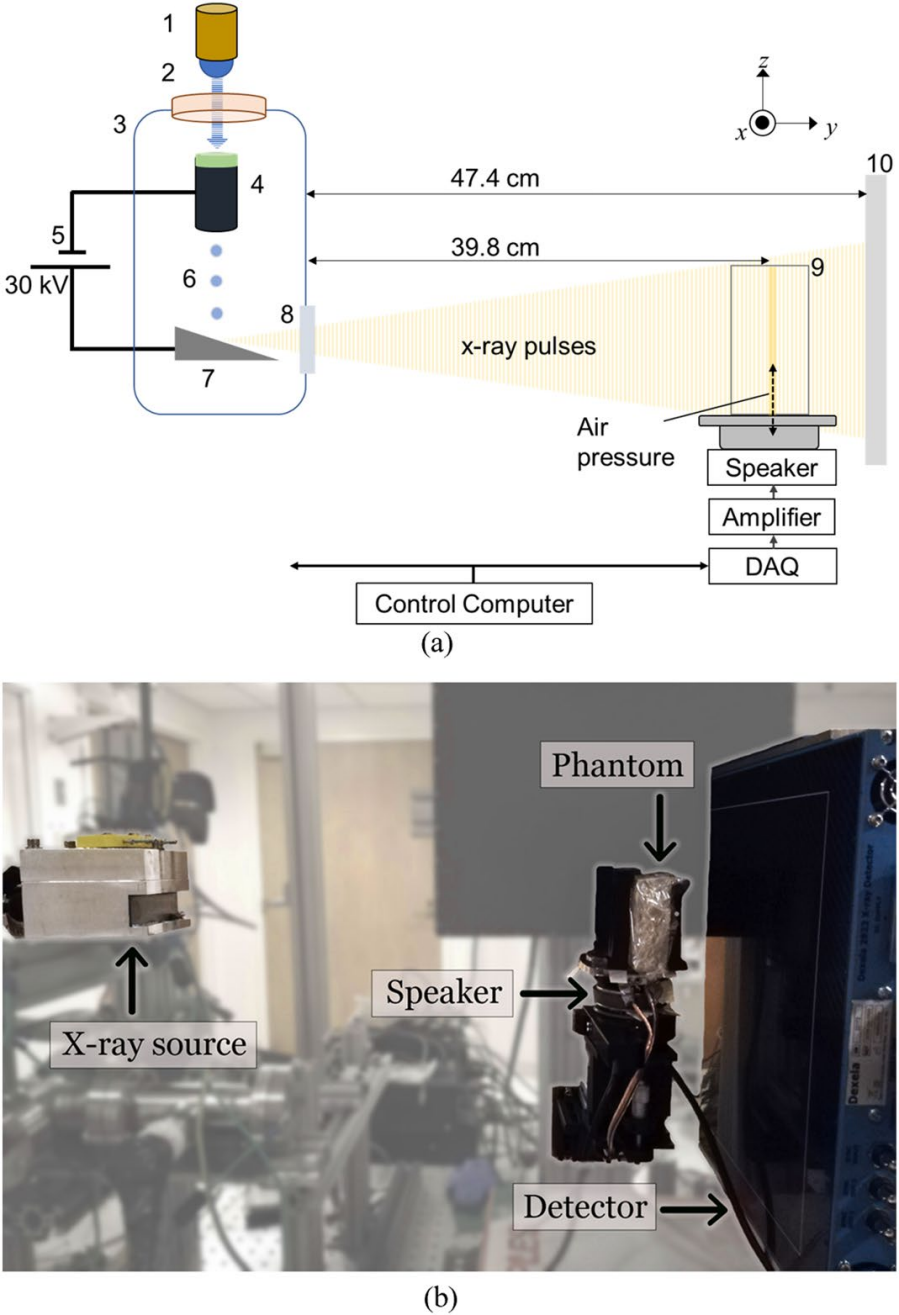
(**a**) A pulsed 255 nm UV LED (1) is used to illuminate a magnesium thin film through a quartz window (2). The thin film, shown in green color, is deposited on a glass electron multiplier (4). The photocathode and anode are both contained within a vacuum manifold (3) pumped down to 10^–7^ Torr by a turbo pump and sealed by a beryllium window (8). The output electrons of the photocathode (6) are accelerated through a high voltage supplied by (5) to a tungsten target anode (7), producing X-ray pulses through the Bremsstrahlung process. These pulses illuminate a phantom (9), depicted in detail in Fig. 3. The phantom is vibrated pneumatically by a speaker synchronized with the UV LED. The images are acquired at different phases of the vibration by a flat-panel detector (10). (**b**) A photograph of the experimental setup.

Figure 1a schematically illustrates the experimental setup for dynamic 2D X-ray elastography using a compact pulsed X-ray source. A photograph of the experimental setup is shown in Fig. 1b. The pulsed X-ray source used in this research has been described previously^23^. The setup consists of an X-ray source, a gel phantom with a vibration stage, a detector for image acquisition, and a control unit. The continuously acquired 2D images of the phantom, under the influence of the vibration stage, are post-processed to compute the stiffness map. The individual components of our setup, and the processing steps for deriving the elasticity or stiffness maps, are described below.

### Pulsed X-ray source

Our X-ray source, which is described in detail in Cramer et al.^24^, uses a pulsed ultraviolet (UV) light emitting diode (LED) emitting at 255 nm. The UV LED is placed outside the vacuum manifold of the X-ray source. The light from this LED strikes a photo-emissive magnesium film inside the vacuum manifold via a quartz window. A small number of photoelectrons generated in this matter are amplified by a Channeltron™ (Photonis Inc., Sturbridge, MA) electron amplifier by a factor of up to 10^9^. The output electron current from the Channeltron™ is then accelerated through a high voltage to strike a tungsten target. The optical spot size of the resulting X-ray focal spot is 4.5 mm (horizontal) × 1 mm (vertical). By adjusting the pulse duration, intensity and duty cycle of the UV LED, it is possible to control the X-ray pulses (and beam current) of the X-ray source. Seven of such X-ray sources are housed in a single module and share a common vacuum manifold. The overall 7-element source is designed to be small and lightweight (approximately 1 kg). The 7 sources span approximately 24 angular degrees. In the current demonstration, we used a single X-ray source to generate a pulsed X-ray beam at 30 kVp and 20 μA tube current. The generated X-ray beam was incident upon a phantom mounted on a pneumatic vibration stage described below.

### Gel phantom and shear wave generation

**Figure 2:**
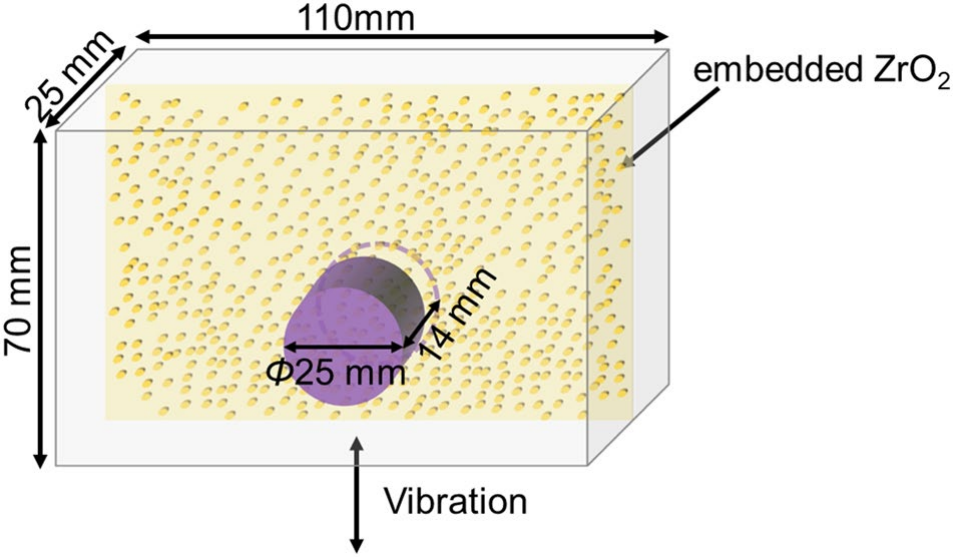
Hitohada gel embedded with ZrO_2_ particles and a 25 mm diameter hard inclusion in the center.

We prepared a Hitohada gel phantom (Fig. 2) from soft urethane resin doped with ZrO_2_ particles. The median grain size distribution of ZrO_2_ was 89 μm, and in this study, 6.3 g of ZrO_2_ particles were spread over the entire phantom when the phantom was in a liquid state. Our phantom had a 25 mm-diameter hard inclusion in the center. We used two types of raw materials of clear Hitohada gel to make the inclusion and the rest of the phantom: H05-100J (EXSEAL Co. Ltd.) was used for the hard inclusion; H00-100J (EXSEAL Co. Ltd.) was used for the surrounding matrix. These materials have a hardness of Asker-C 7 and Asker-C 0, respectively. The hard inclusion simulated a cancerous lesion with a different elasticity but similar radiolucency. The mass attenuation coefficient of the human breast is roughly 0.690 cm^2^/g at an X-ray energy of 20 keV^25^. Assuming a mammary gland density^26^ of 1.02 g/cm^3^, a 2.5 cm thick sample of breast tissue should have an X-ray transmittance on the order of 0.17. The 2.5 cm thick gel phantom used in this experiment had a measured transmittance of 0.15 ± 0.01.

We pneumatically vibrated the phantom using a 8 cm diameter Fostex M800 speaker with an added plastic cover with a 1 mm diameter hole. The sound from the speaker, which induced air pressure wave to vibrate the phantom, was generated by a sinusoidal signal from a data acquisition module or DAQ (National Instruments, USB-6002). The sinusoidal signal from DAQ was amplified using a power amplifier (Bose, Free Space IZA250-LZ) in order to drive the Fostex speaker. The speaker pneumatically vibrated the phantom in the z-direction at a frequency of 115 Hz in order to generate shear waves in the gel. The vibrational acceleration was lower than the limit set by the European Union directive limiting occupational exposure to whole-body and extremity vibrations (2002/44/EC)^27^. With the speaker on, we acquired stroboscopic absorption images at each phase of the vibration to obtain a time-varying, two-dimensional view of the shear wave.

### Synchronized image acquisition

The phantom was illuminated with pulsed X-rays synchronized to the DAQ and the speaker. X-ray images were acquired using a CMOS X-ray flat panel detector (Dexela 2923) that was located 47.4 cm from the X-ray source. The pixel size of the detector was 75 μm × 75 μm. The magnification of the phantom was 1.2 as shown in Fig. 1. Therefore, the effective pixel size at the isocenter of the phantom was 63 μm × 63 μm.

**Figure 3:**
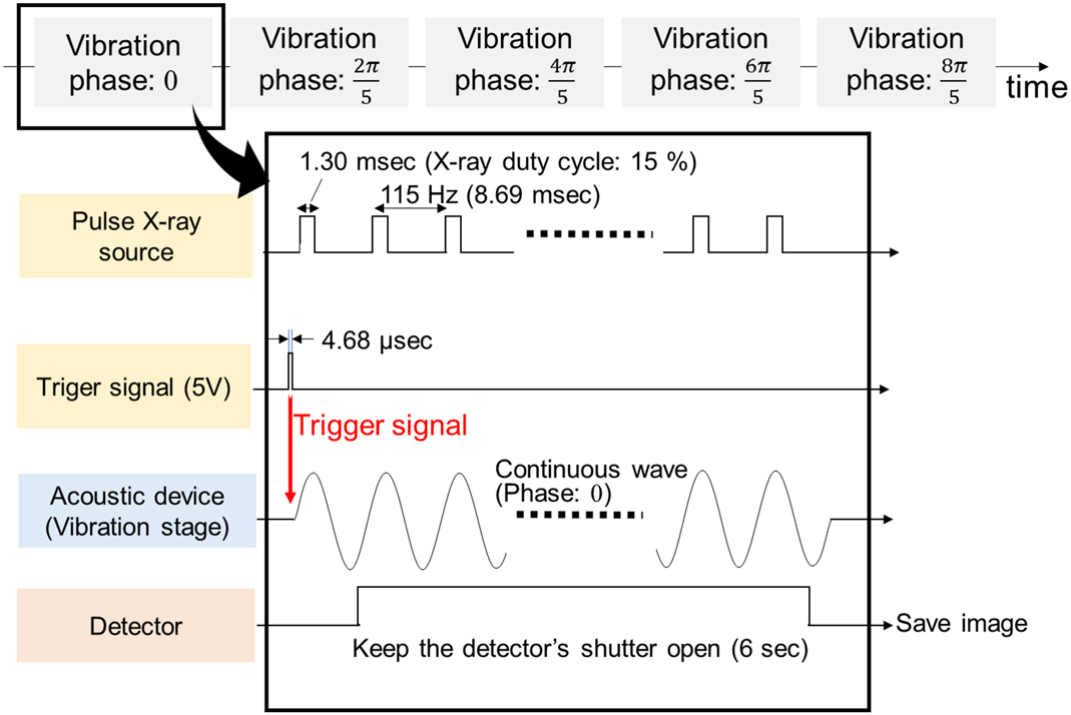
Image acquisition sequence for dynamic X-ray elastography using a pulsed X-ray source. A timing trigger signal synchronizes the vibration stage, X-ray source, and detector image acquisition.

The duty cycle of the pulsed X-ray was 15%, with a pulse width of 1.3 ms as shown in Fig. 3. An X-ray projection image of the phantom was accumulated for 6 s, i.e., the electrical shutter of the detector was kept open for this duration. Therefore, the cumulative time for which the phantom was exposed by X-rays to obtain an X-ray projection image was 0.9 s.

The sinusoidal signal from the DAQ was synchronized with X-ray pulses by a trigger from the pulsed X-ray start timing. We obtained X-ray projection images at five different phases of the air pressure vibration: $0, \frac{2}{5}\pi , \frac{4}{5}\pi , \frac{6}{5}\pi ,\text{ and }\frac{8}{5}\pi$ (i.e., 0, 72, 144, 216, and 288 degrees, respectively) with respect to the vibration timing.

### Image processing and elasticity map generation

To obtain a two-dimensional elasticity map, we used a three-step process that is briefly summarized below^22^.

**Figure 4:**
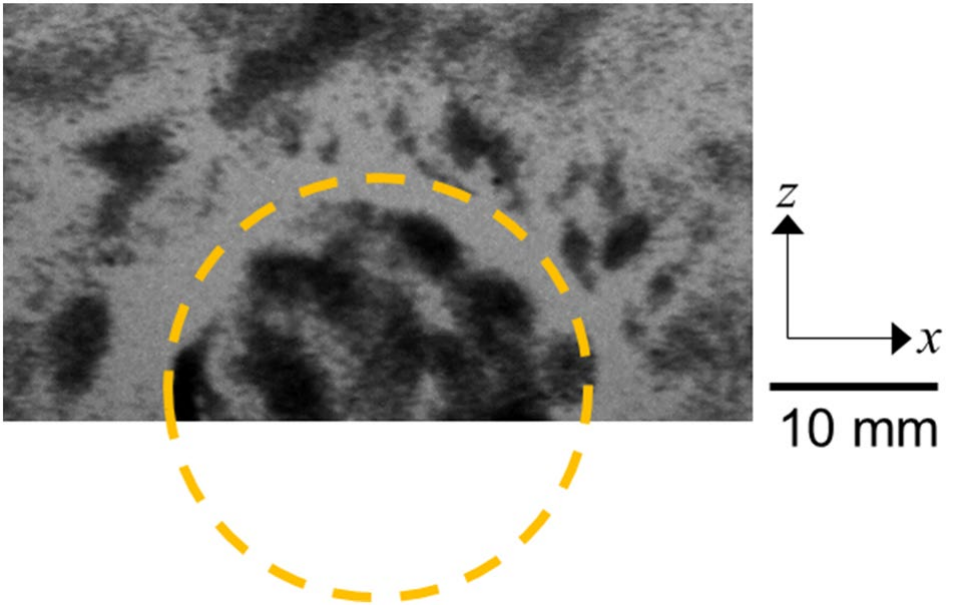
X-ray attenuation image of the phantom showing the hard inclusion (enclosed by a dotted circle) embedded in the surrounding matrix.

First, we obtained the X-ray projection images at the 5 phases $\left(0, \frac{2}{5}\pi , \frac{4}{5}\pi , \frac{6}{5}\pi ,\text{ and }\frac{8}{5}\pi \right)$ as shown in Fig. 4 and illustrated in the Supplementary Movie 1. The portion marked by the dotted circle is the hard inclusion embedded in the surrounding matrix. The region of the phantom close to the vibration source and the region far from the vibration source were not used for data analysis because of the nonlinear behavior of the phantom under large deformation^28^ and low measurement accuracy due to small vibration displacement, respectively.

**Figure 5:**
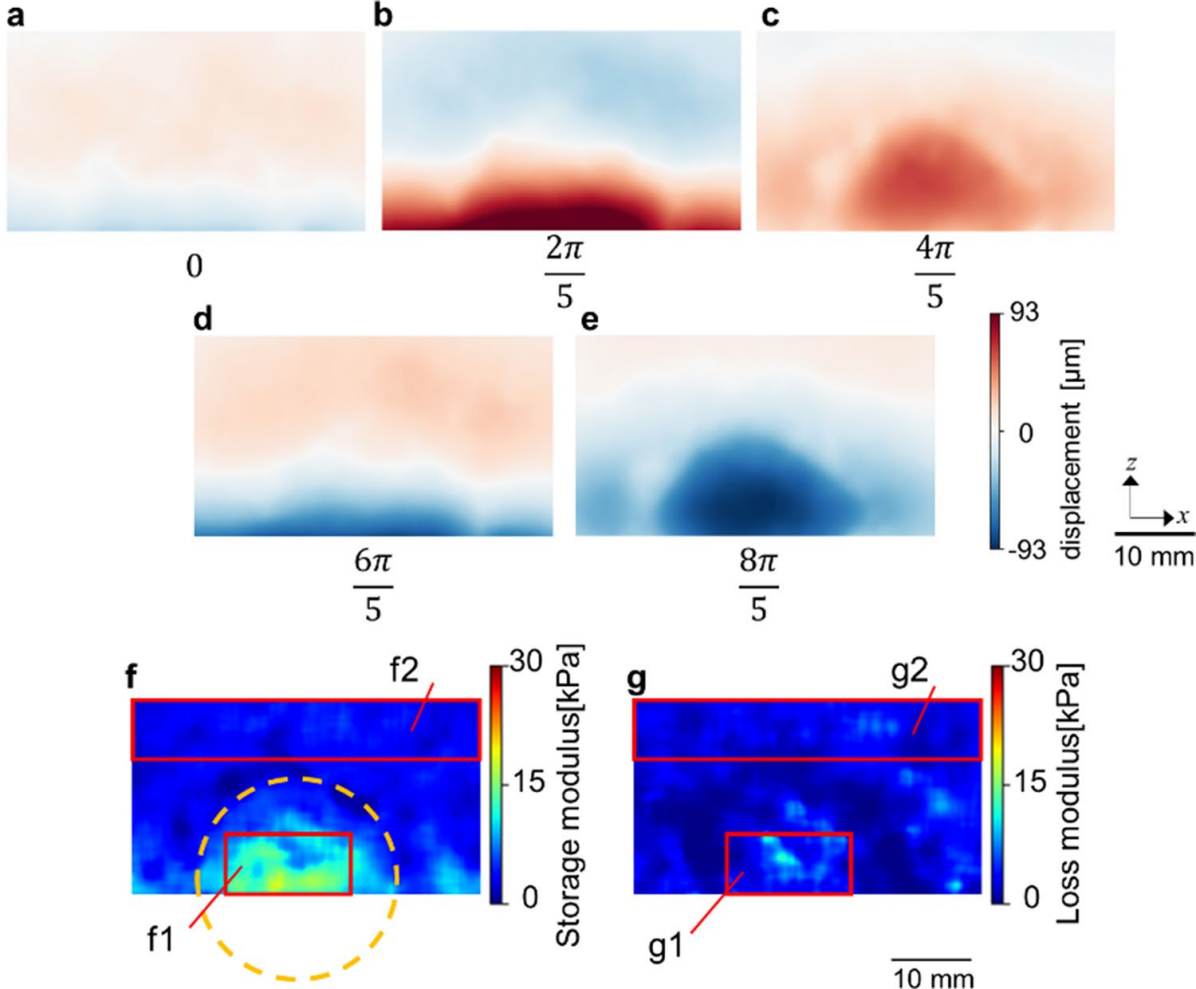
(**a–e**) Displacement maps at phase angles of $0, \frac{2}{5}\pi , \frac{4}{5}\pi , \frac{6}{5}\pi ,\text{ and }\frac{8}{5}\pi$ radians, respectively. (**f,g**) Maps of storage and loss moduli, respectively, for the phantom shown in Fig. 4.

We then obtained displacement maps in the vertical direction (i.e., along the displacement vector of the shear wave) as shown in Fig. 5a–e and the Supplementary Movie 2. We retrieved the displacement at each pixel in the maps by using a non-rigid registration algorithm—a non-parametric diffeomorphic image registration algorithm based on Thirion’s demons algorithm^29^—implemented in MATLAB (Version 9.5.0, The MathWorks, Inc., Natick, MA, USA), and Butterworth bandpass filtering^30^ for denoising. Our non-rigid registration algorithm, which non-linearly accounts for the local distortion field at each pixel, estimates a displacement field that aligns two images. In our case, the image with 0 rad phase was used as the index image, and the distortion field from it to the images with the phase timing of $\frac{2}{5}\pi , \frac{4}{5}\pi , \frac{6}{5}\pi ,\text{ and }\frac{8}{5}\pi$ were computed. We applied the fringe scanning method^31^ to this image set and mapped the displacement from the center of vibration amplitude for each pixel as shown in Fig. 5a–e and the Supplementary Movie 2.

Finally, storage and loss modulus maps for the phantom were reconstructed on the basis of the incompressible algebraic inversion of the differential equation (AIDE)^32^ for an incompressible material ($\nabla \bullet {\varvec{u}}=0$, where ***u*** is the displacement vector). In the AIDE, the complex shear modulus *G* is calculated from the wave equation in a stationary state (the Helmholtz equation) as follows:

1 $$\begin{array}{c}G=-\rho {\omega }^{2}\frac{{U}_{i}}{{\nabla }^{2}{U}_{i}}.\end{array}$$

Here, ρ is density, ω is angular frequency of vibration, ${U}_{i}$ is discrete Fourier transform of ${u}_{i}$ with respect to time, where ${u}_{i}$ ($i=x, y, z$) is components of ***u***. The complex shear modulus can be expressed by $G={G}^{{\prime}}+iG{^{\prime\prime}}$, where ${G}^{{\prime}}\equiv Re\left(G\right)$ and ${G}^{{{\prime\prime}}}\equiv Im\left(G\right)$ corresponding to the storage and loss moduli, respectively. As the Poisson’s ratio of living tissue is between 0.490 and 0.499^33^, we approximate both the phantom material and human tissue as incompressible. The volumetric mass density of Hitohada gel was determined to be 1.0 g/cm^3^. We excluded a small displacement area near the vibration stage. After that, we applied a median filter (25 pixels × 25 pixels) to the storage and loss modulus maps to make it smooth.

## Results

Figure 5f,g shows the maps of the storage and loss moduli obtained from Fig. 5a–e. As can be seen, the high storage modulus region corresponds to the region designed to simulate the cancerous lesion; this region has higher elasticity and is denoted by a dotted circle in Fig. 5f. Therefore, our approach is able to distinguish the hard inclusion in a matrix. The storage moduli of the square regions in Fig. 5f denoted (f1) and (f2) were 12 ± 2.5 kPa and 3.4 ± 0.78 kPa, respectively. The loss moduli in the corresponding squares denoted by (g1) and (g2) in Fig. 5g were 4.4 ± 2.1 kPa and 2.8 ± 1.0 kPa, respectively.

We calculate of the contrast noise ratio (CNR) as follows:

2 $$\begin{array}{c}CNR=\sqrt{\frac{2{\left(\overline{S }-\overline{B }\right)}^{2}}{{{\sigma }_{s}}^{2}+{{\sigma }_{B}}^{2}}}.\end{array}$$

Here, $\overline{S }$ and $\overline{B }$ are the mean of the signal and background, ${\sigma }_{s}$ and ${\sigma }_{B}$ are the standard deviation of the signal and background^34^. “S” is the value measured in the areas of (f1) and (g1) in Fig. 5 and “B” is the value measured in (f2) and (g2) in Fig. 5. The CNR in the storage modulus from (f)-1 to (f)-2 was 4.5. On the other hand, the CNR in the transmission image from the same ROI of (f)-1 to (f)-2 was 1.1. As such, the elastography outperforms naïve X-ray transmission imaging in its ability to discriminate a hard inclusion from surrounding material.

## Discussion

We demonstrated two-dimensional dynamic X-ray elastography by applying pneumatic vibration to a Hitohada gel phantom. Maps of storage and loss moduli were computed from the wave equation of the shear waves as they propagated through the phantom. The deformations imparted by the shear wave were imaged with a net exposure time of 4.5 s and an effective pixel size of 62 μm. The storage modulus map, obtained from dynamic elastography, was able to distinguish a hard inclusion inside a matrix with a CNR of 4.5. This is substantially better than the 1.1 CNR between these regions in the pure X-ray attenuation images. We used two types of raw materials of clear Hitohada gel to make the inclusion and the rest of the phantom: H05-100J (EXSEAL Co. Ltd.) was used for the hard inclusion; H00-100J (EXSEAL Co. Ltd.) was used for the surrounding matrix. We measured these two types of materials of clear Hitohada gel using a rheometer (Anton Paar, MCR302) at a vibration frequency of 16 Hz (Table 1, left). The obtained storage moduli were of the same order as breast cancer and normal breast tissue^35, 36^ (Table 2). The overall setup, using a pulsed X-ray source, was markedly more compact as compared to our previous setup that used a continuous X-ray with a chopper wheel.

**Table 1:** Mechanical properties of the Hitohada phantom.

Phantom	Rheometer	X-ray elastography
	Storage modulus (kPa)	Storage modulus (kPa)
Harder inclusion H05-100J (EXSEAL Co. Ltd.)	9.7 ± 1.7	12 ± 2.5
Surrounding matrix H00-100J (EXSEAL Co. Ltd.)	4.3 ± 1.5	3.4 ± 0.78

**Table 2:** Mechanical properties of the breast tissue.

Mammary gland	MR elastography^33^	US elastography^34^
	Median elasticity (kPa)	Shear elasticity (kPa)
Mammary gland cancer	15.9 (malignant invasive breast tumors)	16.76 ± 13.10 (malignant masses)
Normal tissue	7 (benign breast lesions) 2.5 (breast parenchyma)	1.40 ± 1.12 (benign masses)

The storage and loss moduli were calculated by experiments at a single vibration frequency. In the future, using the results of vibration with multiple frequencies will allow us to examine how viscosity and elasticity are combined using a model such as the Kelvin-Voigt or Maxwell model^37–39^. Being able to examine viscosity and elasticity of tissues together may allow for more detailed study of disease progression.

The present paper focused on 2D elasticity maps. Because we used projection X-ray images of the breast phantom, any depth information along the direction of the X-ray projection is lost. Therefore, the elasticity maps are an aggregate, representing the whole thickness of the phantom. For many applications in medical diagnostics, including breast imaging, it is important to retain the depth information. In breast imaging, this is accomplished using tomosynthesis^40^, an imaging technique in which a volumetric image is obtained from a series of projection images acquired along an angular span. Digital tomosynthesis has rapidly expanded in clinical practice and is the preferred method for evaluation of the breast.

The experimental setup and the image processing methods presented in this paper have the potential to realize X-ray elastography and tomosynthesis in a volumetric fashion. Tomosynthesis with the source module used in this study is feasible by virtue of the fact that the X-ray source has 7 X-ray elements spanning 24 degrees. Therefore, using 3 such sources side-by-side, one can create a 21-element arc of sources spanning approximately 72 degrees. These sources can be electronically steered, and such an assembly can be used for X-ray tomosynthesis without any moving parts. With the help of a vibration stage, it is possible to obtain the projection images over 4 or 5 different phases of shear wave propagation through the tissue. These projection images can then be converted into multiphase tomosynthesis slices, which can then be used to produce slice-by-slice elasticity maps. All imaging for yielding slice-by-slice elasticity maps can be acquired without any moving parts in the setup and without rotating or displacing the sample.

One limitation of the study is the relatively large size (25 mm) of the tumor model used in this experiment. Further research is required to determine the performance of X-ray elastography in identifying smaller tumors.

**Figure 6:**
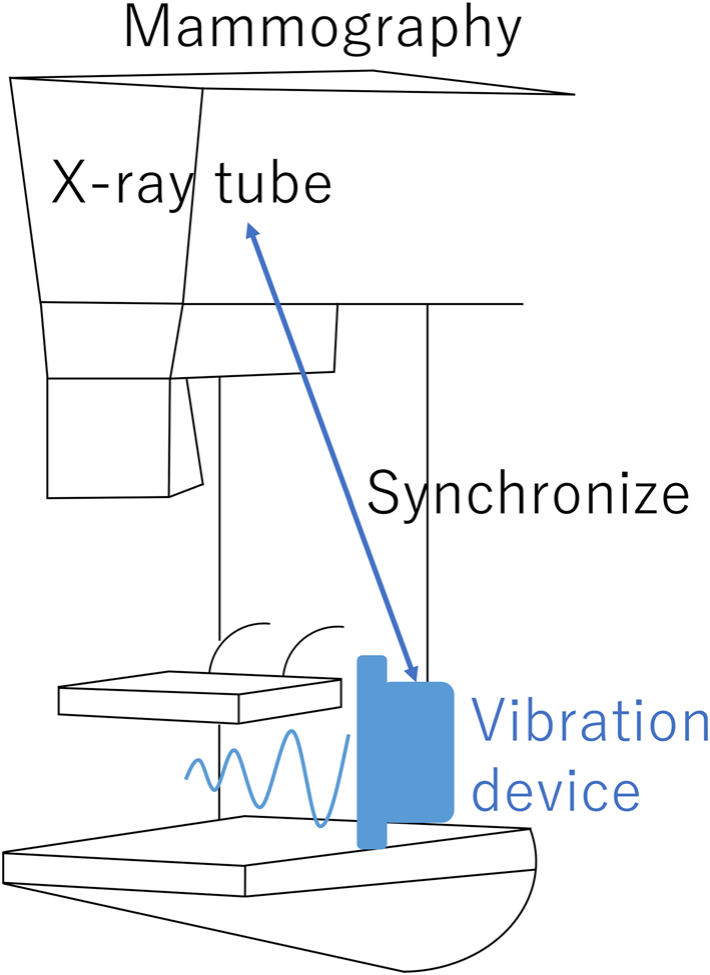
Proposed implementation geometry.

The mean glandular dose (MGD) of full-field Digital Mammography (FFDM) and Digital Breast Tomosynthesis (DBT) for are approximately 1.4 mGy and 1.9 mGy, respectively, for the craniocaudal views as well as the mediolateral oblique views^41^. We calculate that X-ray elastography can be performed at doses below the European Union and International Atomic Energy Agency MGD limit of 2.5 mGy^42^. The calculated doses were 0.45 mGy for a tissue thickness of 2.5 cm, as in this experiment, and 0.91 mGy for a clinical mammography exam assuming a tissue thickness of 4 cm. These values were calculated by assuming that the conversion efficiency of the X-rays is 1% of the input, assuming that the X-rays emitted from the target spread out in a spherical shape, ignoring the effects of X-ray absorption and scattering by air, and also ignoring the effects of intensity uniformity in the irradiation field. For X-ray elastography, one can divide this dose budget into lower dose projections that are timed and synchronized with the acoustic vibration according to the schedule described in this paper. As a result, the acquisition of the additional stiffness properties of the breast can essentially be dose neutral both in the FFDM and DBT setups. A possible implementation is depicted in Fig. 6.

Another possible application of this technique is compact X-ray elastography of small animals for basic medical science. This small and cost-effective X-ray elastography method would yield higher spatial resolution, deeper tissue penetration, and lower costs.

## Supplementary Information

Supplementary Legends.

Supplementary Video 1.

Supplementary Video 2.
