# Supplementary material for: Dynamic X-ray elastography using a pulsed photocathode source
Source: Sci Rep. 2021 Dec 16;11:24128. doi: 10.1038/s41598-021-03221-y (PMC8677743; doi:10.1038/s41598-021-03221-y)
Supplement: Supplementary file 1 — Supplementary Legends. [file 41598_2021_3221_MOESM1_ESM.pdf]

Author list, Dynamic X-ray Elastography using a Pulsed Photocathode Source

Chika Kamezawa[1, 2, 3],

Avilash Cramer[4, 5],

Wolfgang Krull[6],

Wataru Yashiro[3],

Kazuyuki Hyodo[1, 2],

and Rajiv Gupta[5, 6*]

[1] Department of Materials Structure Science, SOKENDAI (The Graduate University for Advanced Studies), 1-1 Oho, Tsukuba, Ibaraki 305-0801, Japan

[2] Institute of Materials Structure Science, High Energy Accelerator Research Organization (KEK), 1-1 Oho, Tsukuba, Ibaraki 305-0801, Japan

[3] Institute of Multidisciplinary Research for Advanced Materials (IMRAM), Tohoku University, 2-1-1 Katahira, Aoba-ku, Sendai, Miyagi 980-8577, Japan

[4] Massachusetts Institute of Technology, Cambridge, 02139, USA

[5] Harvard Medical School, Boston, 20115, USA

[6] Massachusetts General Hospital, Boston, 02114, USA

Supplementary movie legends

Supplementary movie 1

X-ray attenuation images for the phantom at five phases.

Supplementary movie 2

Displacement maps for the phantom at five phases.
